# Genomic insights on cgMLST markers, drug resistance, and urease cluster of *Proteus mirabilis* strains

**DOI:** 10.1128/spectrum.00992-24

**Published:** 2024-12-06

**Authors:** Shitao Lian, Yadong Liu, Songnian Hu, Chen Shen, Yinping Ma, Peng Yin, Zilong He

**Affiliations:** 1 School of Engineering Medicine, Beijing Advanced Innovation Center for Big Data-Based Precision Medicine, Interdisciplinary Innovation Institute of Medicine and Engineering, Beihang University, Beijing, China; 2 State Key Laboratory of Microbial Resources, Institute of Microbiology, Chinese Academy of Sciences, Beijing, China; 3 University of Chinese Academy of Sciences, Beijing, China; 4 Department of Urology, The Second Affiliated Hospital of Dalian Medical University, Dalian, Liaoning, China; 5 CapitalBio Technology, Beijing, China; Beijing Institute of Genomics, Chinese Academy of Sciences, Beijing, China

**Keywords:** *Proteus mirabilis*, cgMLST markers, drug-resistance, urease gene cluster, plasmid genome prediction

## Abstract

*Proteus mirabilis*, a significant pathogenic bacterium within the Enterobacteriaceae family, is widely distributed across various natural environments. This study conducted a genomic comparison analysis of 1,267 strains of *P. mirabilis* using extensive genome data from public databases. The objective was to elucidate the pan-genomic structure of *P. mirabilis*, revealing the composition and distribution of core and accessory gene families among different strains. Additionally, an attempt was made to construct a core genome multilocus sequence typing scheme specific to this species in order to enhance the precision of describing genetic diversity and evolutionary relationships. Furthermore, the study delved into the mechanisms of resistance of *P. mirabilis* to carbapenems and quinolones. Our findings underscore significant challenges posed by *P. mirabilis* in terms of antibiotic resistance, with widespread resistance observed particularly against beta-lactams and an increasing trend in resistance to carbapenems and quinolones. These results highlight the severity of *P. mirabilis* as a pathogen and underscore its rapid evolution and adaptability in developing resistance. This study aims to deepen our understanding of the antibiotic resistance of *P. mirabilis*, providing important insights for the development of future antimicrobial drugs, promoting effective treatment and control of this pathogen, and mitigating its threat to human health.

**IMPORTANCE:**

The bacterium *Proteus mirabilis* is a common pathogenic bacterium that is known to cause a variety of human infections. The drug-resistant genes carried by *P. mirabilis* present a significant challenge to clinical treatment, particularly in regard to the organism’s notable resistance to commonly used beta-lactam and quinolone drugs. Furthermore, the prevalence of the urease gene cluster of *P. mirabilis* at the urease gene level may be associated with the formation of kidney stones. The objective of the study is to analyze the bacterium’s drug resistance, urease gene clusters, and gene distribution in genomes in order to facilitate the development of antimicrobial drugs and improve the treatment and control of *P. mirabilis* infections.

## INTRODUCTION

*Proteus mirabilis* is a significant pathogenic bacterium, characterized by its unique ability for migratory growth and population differentiation (1). As a Gram-negative bacterium, *P. mirabilis* is widespread in nature and can be isolated from a variety of ecological niches such as soil, water bodies, rubbish, decaying organic matter, and the intestinal tract of humans or animals (2). This species is capable of causing a variety of human infections, including wound infections, eye infections, gastrointestinal infections, and urinary tract infections (3, 4). *P. mirabilis* is one of the most common pathogens in hospital-acquired infections, especially in patients who have been hospitalized for long periods or who use medical devices such as catheters (5, 6). It has been reported that infections cause between 20% and 40% of all urinary tract stones. Urinary stones are a common trigger for urinary tract infections caused by *P. mirabilis*. Urinary tract infections caused by *P. mirabilis* may lead to a series of complications, including bladder and kidney stones, acute pyelonephritis, and bacteremia, which can pose a serious threat to the patient’s health and even endanger their life (7–9).

*P. mirabilis* has a potent urease activity that can hydrolyze urea into ammonia and carbon dioxide (10, 11). It leads to the precipitation of soluble ions in the urine and the eventual formation of guano and carbonate apatite stones. Bacterial urease is an enzyme encoded by the structural genes *ureA*, *ureB*, and *ureC* and the accessory genes *ureD*, *ureF*, *ureG*, and *ureE* (12, 13). Bacterial urease is involved in the hydrolysis of urea during human metabolic processes, and when the environment is alkaline, the products of urea hydrolysis will undergo crystalline precipitation and eventually form infected stones (14).

*P. mirabilis* has been reported to carry a variety of resistance genes, the main common being beta-lactams as well as quinolones (15). The beta-lactamases produced by bacteria are usually classified into the types of penicillinases, cephalosporinases, broad-spectrum beta-lactamases, and ultrabroad-spectrum beta-lactamases (16, 17). The carbapenemases belong to the ultrabroad-spectrum enzyme class of beta-lactamases, and they are capable of breaking down a wide range of beta-lactam antibiotics, including carbapenems. These enzymes are particularly prevalent in *P. mirabilis*, where the major enzyme types include KPC-2, NDM, and VIM (18–20). The mechanism of resistance to quinolone antimicrobials in *P. mirabilis* mainly involves mutations in the quinolone-resistant region of the chromosome, which is caused by point mutations in the *gyrA*, *gyrB*, and *parC* genes (21, 22). In addition, resistance to quinolone antimicrobials in *P. mirabilis* is also closely related to the resistance-nodulation-cell division (RND) antibiotic efflux pump mechanism (23–25).

With advances in next-generation sequencing technology, the genomic studies of *P. mirabilis* are increasing. For example, Potter et al. performed a comparative genomic analysis of 893 clinical strains of *P. mirabilis*, revealing the genomic diversity of the species as well as several genomic features (26). Saeb et al. performed pathogenetic genomics and resistance analyses of a specific isolate of SCDR1, which was found to have a variety of drug resistance mechanisms (27). Despite the increasing number of genomic studies on *P. mirabilis*, the overall drug resistance and urease of this species at the population genome level are still insufficient and need to be further explored.

In this study, we conducted a pan-genomic, resistance genomic, and urease comparative genomic study of *P. mirabilis* (1,267 strains) using data from public databases to reveal the overall drug resistance, composition, and distribution of urease genes cluster. The objective of this study is to elucidate additional information regarding the drug-resistance mechanism exhibited by *P. mirabilis* and to explore the mechanism of urease using bioinformatic tools in order to anticipate genetic modifications and mutations. We anticipate that our findings will make valuable contributions to the advancement of effective strategies aimed at mitigating drug resistance in *P. mirabilis*.

## MATERIALS AND METHODS

### Public genome data download and analysis

To visually illustrate the research methods and analytical processes, we have created a flowchart depicting the study workflow as shown below (Fig. S1). We acquired publicly available genomes of *P. mirabilis* (*n* = 1,311) along with associated metadata from the NCBI database (https://ncbi.nlm.nih.gov/). To evaluate the completeness and quality of these sequences, we utilized BUSCO (28) (version 5.4.6) and QUAST (29) (version 5.2.0). Subsequently, we selected 1,267 high-quality genomes for further analysis after rigorous quality control. Gene annotation of the *P. mirabilis* genomes was conducted using Prokka (30) (version 1.14.6).

### Pangenome and phylogenetic tree analysis of *P. mirabilis*

We employed 1,267 strains of *P. mirabilis* in GFF format obtained from Prokka for pan-genome analysis using Roary (31) (version 3.13.0). Subsequently, pan-genome and core genome maps were generated using the create_pan_genome_plots.R script. The core genome multiple alignment produced by Roary was used to construct a core genome multilocus sequence typing (cgMLST) (32) phylogenetic tree with FastTree (33) (version 2.1.11), which was visualized using the iTOL (interactive tree of life) website (34) (https://itol.embl.de/). Two-by-two genome comparison ANI values were obtained using FastANI (35) (version 1.34) and visualized as heatmaps.

### Drug-resistant genes prediction of *P. mirabilis*

We employed Resistance Gene Identifier (36) (perfect, stringent mode; version 5.1.1) to predict antibiotic resistance genes from the coding sequences of 1,267 *P*. *mirabilis* strains against the Comprehensive Antibiotic Resistance Database (https://card.mcmaster.ca/). Subsequently, statistical and functional analyses were conducted on the predicted resistance genes, and their visualization was achieved using heatmaps.

### Plasmid sequences prediction of *P. mirabilis*

We conducted plasmid sequence prediction for 1,267 *P*. *mirabilis* strains using Plasmer (37) (version 23.04.20). Subsequently, we integrated these data with previously predicted drug resistance information, resulting in the identification of 8,133 plasmid genomes associated with drug resistance in *P. mirabilis*. Finally, we utilized the Proksee (38) online tool (https://proksee.ca) for annotation and visualization of the plasmid genomes.

## RESULT

### Basic information of *P. mirabilis* genomes

We downloaded 1,311 genome sequences of *P. mirabilis* from the NCBI database. We evaluated the completeness and quality values of these genome sequences with Busco and Quast and finally selected 1,267 high-quality sequences (completeness > 95% and contigs number < 1,000). Among the high-quality strains, we found that the number of contigs ranged from 1 to 688, and the maximum value of N50 was 4,372,742 bp and a minimum value of 10,660 bp, with an average genome size of about 4,021,165 bp. The above 1,267 genomes were annotated by Prokka, in which the average number of mRNAs per strain was 3,735. Based on the above analysis, we believe that these genomes are qualified and can be analyzed subsequently (Table S1).

### Pan-genomic analysis and phylogenetic tree construction based on cgMLST markers in *P. mirabilis*

We performed a pan-genomic analysis of *P. mirabilis* (Table S2[A]). We found that the *P. mirabilis* genomes contained 43,940 gene families, of which there were 2,093 core genes (frequency ≥ 95%), accounting for 4.76%, 2,273 shell genes (15% ≤ frequency < 95%), and 39,574 cloud genes (0% ≤ frequency < 15%), accounting for 5.17% and 90.06%, respectively. The pan-genome map of *P. mirabilis* (Fig. S2) indicates an open conformation, with its size expanding as more genomes are analyzed. This expansion is directly linked to the increasing number of emerging gene families within the pangenome.

Since there is no ST typing in *P. mirabilis* genomes, we developed the cgMLST genes of this species. Based on the cgMLST markers (Table S2[B]), we constructed the phylogenetic tree of 1,267 *P*. *mirabilis* genomes. The tree showed that there was no obvious clade within the population, as shown in Fig. S3, and some of the strains were more widely distributed in continents and had higher diversity. Subsequently, we analyzed 1,267 genomes by ANI (Fig. S4), and the hierarchical clustering based on ANI values showed that there were three subspecies of *P. mirabilis*, and the intragroup similarity was more than 98%. However, the origin of strains in the different clades based on ANI is also diverse and not significantly clustered.

### Drug-resistant genes prediction in *P. mirabilis*

**Fig 1:**
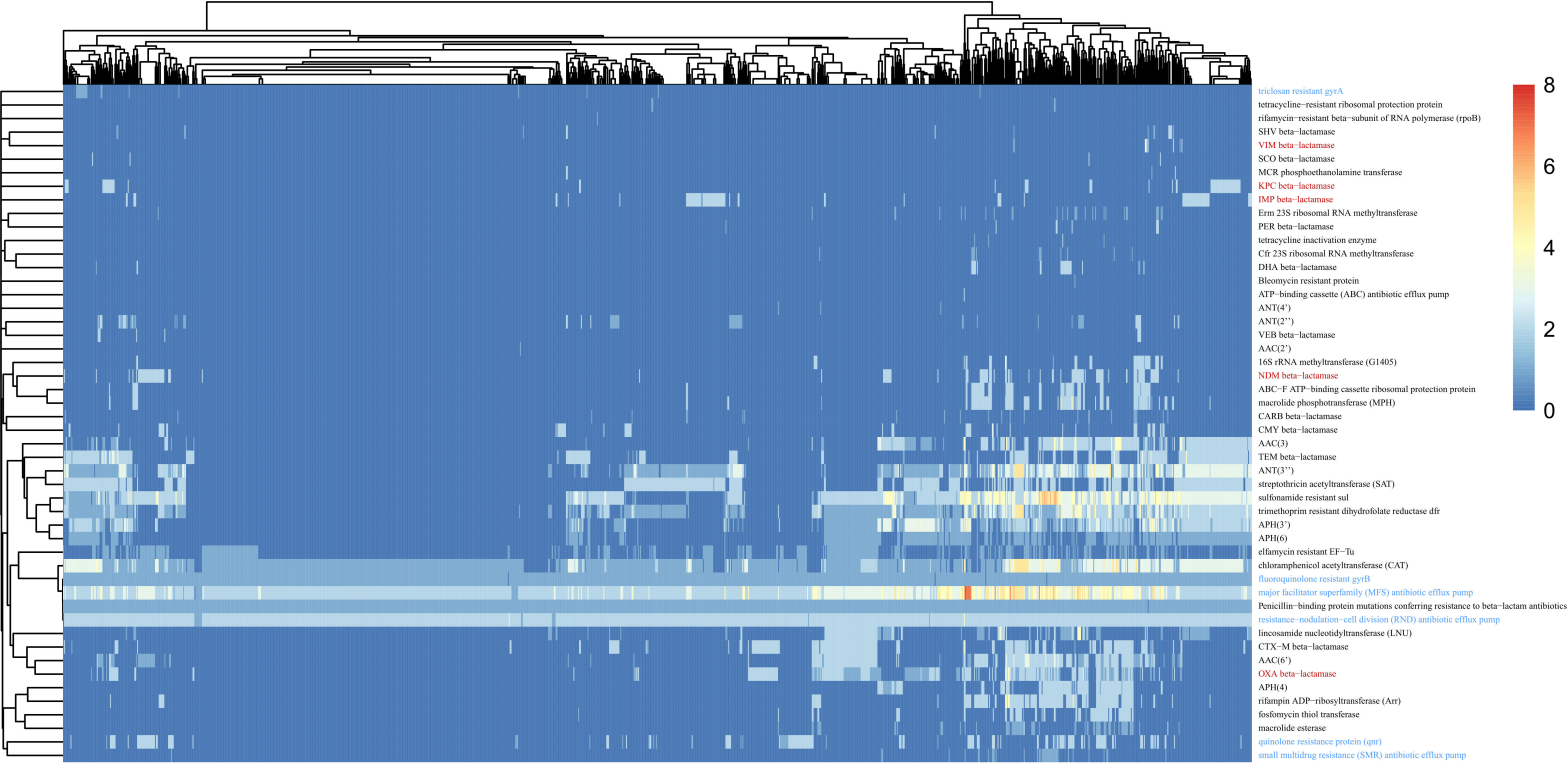
Heatmap of 50 potential drug-resistance genes in *P. mirabilis*. In 1,267 *P*. *mirabilis* strains, antibiotic resistance genes were predicted using RGI (version 5.1.1), drug resistance patterns were analyzed using statistical and functional methods, and the abundance information was visualized through heatmaps. In the figure, two types of highlighted annotation font colors are used, with red font indicating the type of carbapenem-resistance mechanism and blue font indicating the type of quinolone antibiotic resistance mechanism.

We obtained 50 kinds of potential drug-resistant genes profiling of *P. mirabilis* by RGI. As shown in Fig. 1, the proportion of beta-lactam antibiotic resistance was as high as 57.46% (Table S3), of which, carbapenems as four-generation beta-lactam antibiotics likewise showed ultra-high frequency. Among the 1,267 genomes of *P. mirabilis*, the total percentage of carbapenem antibiotic resistance was as high as 29.50% for NDM beta-lactamase, IMP beta-lactamase, OXA beta-lactamase, KPC beta-lactamase, and VIM beta-lactamase, among which NDM beta-lactamase (NDM-1,5,7) had the highest proportion of about 9.55%, IMP beta-lactamase (IMP-4,6,27) and OXA beta-lactamase (OXA-23,48,58) were the next highest, accounting for 7.50% and 6.70%, respectively, and KPC beta-lactamase (KPC-2,3,6) and VIM beta-lactamase (VIM-1,4) with 5.05% and 0.63%, respectively (Table 1).

**TABLE 1:** Prevalence of carbapenem-resistance genes among 1,267 strains of *P. mirabilis*

Carbapenem-resistant organisms	No. of strain	Detection rate
OXA beta-lactamase (OXA-23,48,58)	86	6.70%
NDM beta-lactamase (NDM-1,5,7)	121	9.55%
IMP beta-lactamase (IMP-4,6,27)	95	7.50%
KPC beta-lactamase (KPC-2,3,6)	64	5.05%
VIM beta-lactamase (VIM-1,4）	8	0.63%
Total count (de-duplicated)	374	29.50%

Subsequently, we predicted quinolone-resistant genes based on the RGI results. According to the previous reports (39–41), point mutations (*gyrA*, *gyrB*, and *parC* genes) and plasmid-mediated genes (*qnrA*, *qnrC*, *qnrD*, *qnrS*, and *aac(6')-Ib-cr*) can produce quinolone resistance. There are several major mechanisms of quinolone resistance in our study, as shown in Table 2, with the most common being the *gyrB* point mutation resulting in quinolone resistance. Point mutations on *gyrB* associated with quinolone resistance are the substitution of aspartic acid for glutamic acid at position 466 and the substitution of serine for tyrosine or phenylalanine at position 464, with a total count of 309 occurrences. In 1,267 strains of *P. mirabilis*, there were 20 mutations in the chromosome *gyrA* gene, of which 18 mutations were closely associated with quinolone resistance. These mutations include the substitution of serine for isoleucine or arginine at position 83 and the substitution of glutamic acid for lysine at position 87. In addition, no *parC* gene mutations associated with quinolone resistance were detected in the RGI results. We also found that *qnrA*, *qnrS*, *qnrD*, and *aac(6')-Ib-cr* accounted for 0.24%, 2.4%,10.7%, and 16.5%, respectively. In 1,267 strains of *P. mirabilis*, the *aac(6')-Ib-cr* (*N* = 333), *qnrA* (*N* = 6), and *qnrD* (*N* = 156), *qnrS* (*N* = 57) genes were located on plasmids, and another 54 *aac(6')-Ib-cr* genes were located on chromosomes (Table S4).

**TABLE 2:** Prevalence of quinolone-resistance determinants among 1,267 strains of *P. mirabilis*

Quinolone resistance	No. of strain	Detection rate
Triclosan-resistant gyrA	20	1.58%
Fluoroquinolone-resistant gyrB	1,263	99.68%
Quinolone-resistance protein (qnr)	178	14.05%
RND antibiotic efflux pump	1,267	100.00%
Major facilitator superfamily antibiotic efflux pump	1,266	99.92%

### Urease gene cluster genome structure and evolutionary tree analysis

**Fig 2:**
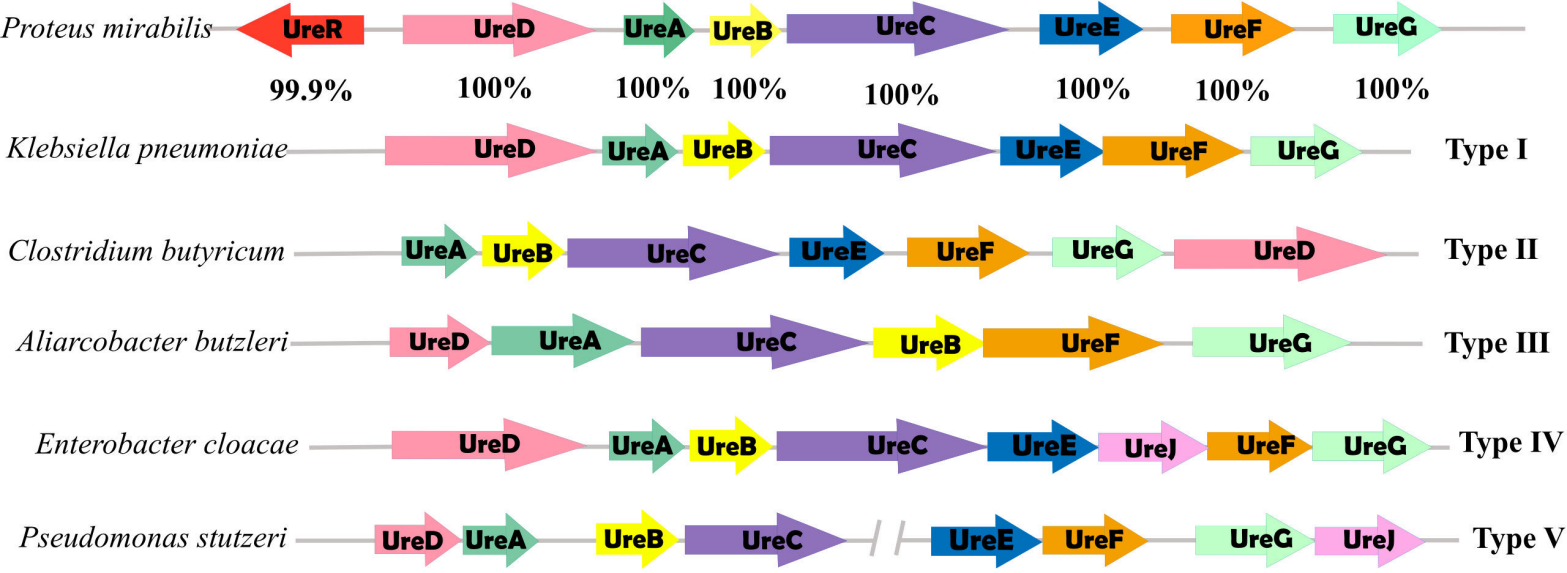
Structure of urease gene clusters in *P. mirabilis* compared to other bacteria. The illustration depicts a comparative analysis of urease gene clusters across various bacterial taxa, represented by an array of gene maps with arrows indicating the direction of transcription. Unique color coding facilitates the identification of individual genes within each cluster. The clusters adjacent to the gene sequences are categorized into Types I through V based on their compositional and sequential structure.

We revealed that urease genes usually exist in the form of gene clusters in the genome of *P. mirabilis*, which contains three structural genes (*ureA*, *ureB*, and *ureC*) and other auxiliary genes (*ureD*, *ureE*, *ureF*, and *ureG*) and regulatory genes (*ureR*). We found that the urease gene clusters of *P. mirabilis* belong to class I of the five types of urease gene clusters reported (42), as shown in Fig. 2. In addition to containing the structural genes (*ureA*, *ureB*, and *ureC*) and auxiliary genes (*ureD*, *ureE*, *ureF*, *ureG*, etc.) that are already present in class I. *P. mirabilis* also added regulatory genes (*ureR*). What’s more, the copy number of urease gene cluster in *P. mirabilis* revealed that some genes have multiple copies in one genome, such as *ureB* and *ureF*.

We also constructed an evolutionary tree of urease by using the single-copy genes (*ureA*, *ureC*, *ureD*, and *ureE*). As shown in Fig. S5, we figured out that there are three obvious clusters in the single-copy consistent evolutionary tree. Then, we further screened the drug resistance information with the tree. We found that the strains that are associated with carbapenem resistance or quinolone resistance are dispersed and not significantly enriched in the three clusters.

### Plasmid genome prediction and analysis of *P. mirabilis*

**Fig 3:**
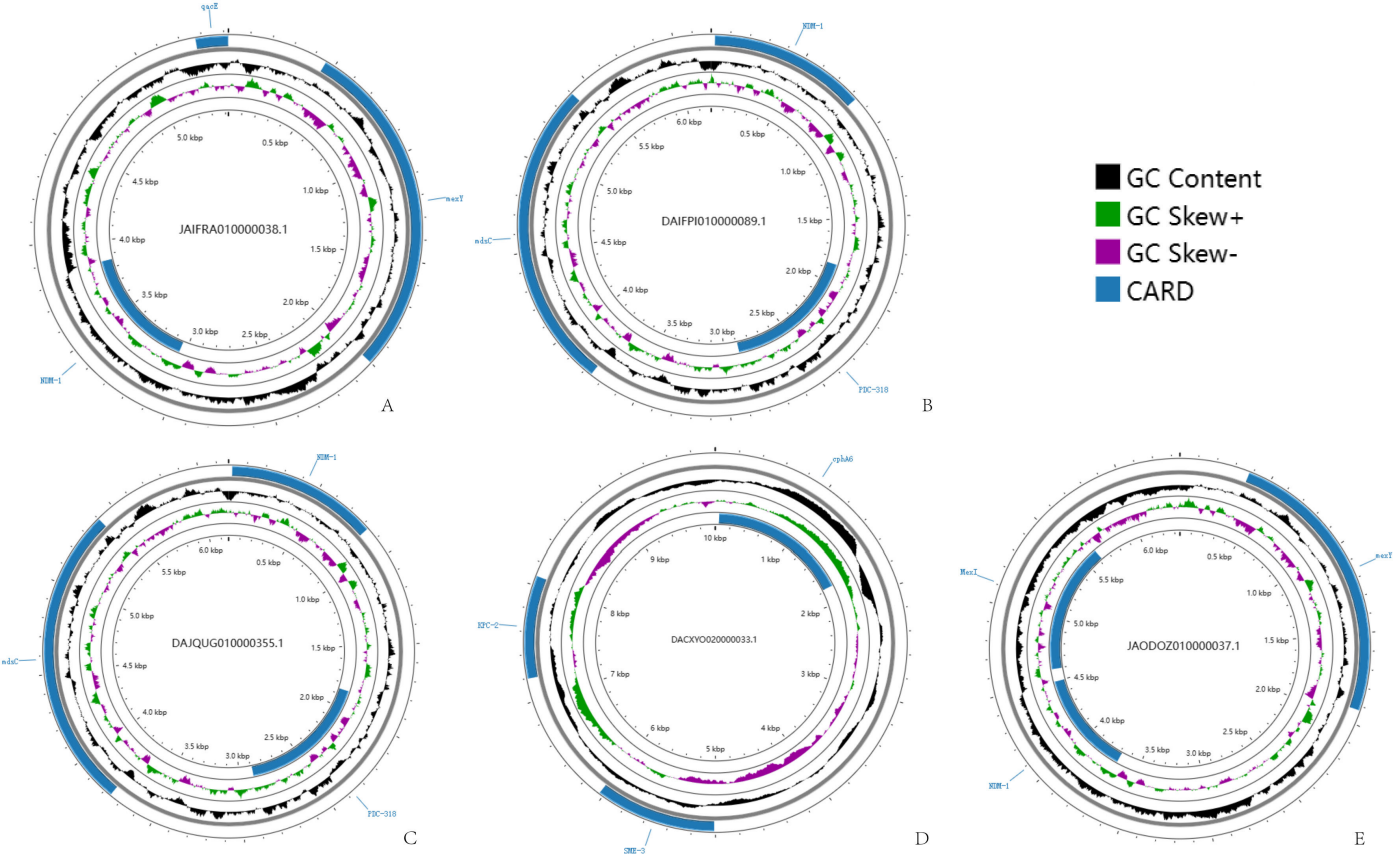
The circular representation map of plasmid. The image presents circular charts of bacterial plasmids annotated with drug-resistance genes. In **A–D**, two types of drug resistance are denoted: carbapenem resistance (NDM beta-lactamase) and quinolone resistance. **(E)** Depicts solely the carbapenem resistance.

We predicted the plasmid sequences by Plasmer and finally screened 8,133 plasmid sequences associated with drug resistance (Table S5). Among them, 210 plasmids were related to carbapenem resistance, and 554 plasmids were related to quinolone resistance. As shown in Fig. 3, we selected five representative plasmids for sequence genome visualization, in which two types of drug resistance both carbapenem resistance (NDM beta-lactamase) and quinolone resistance in Fig. 3A through D and carbapenem resistance in Fig. 3E. Statistically, the genome sizes of each plasmid genome (A–E) are 5,420, 6,195, 6,163, 10,001, and 6,343 bp, and their GC contents were 51.4%, 65.0%, 65.1%, 61.7%, and 54.2%, respectively. In this study, we validated all plasmids based on the NCBI NR database. One originated from *P. mirabilis*, while the rest were from other species (*Escherichia coli* and *Pseudomonas aeruginosa*). Two plasmids were identified as having the same type (*Escherichia coli* strain 190 plasmid unnamed1)(Table 3). Further analysis revealed that out of the five plasmids, three were classified as p6061604, pPA2047, and pHFK418, while the remaining two plasmids were unnamed.

**TABLE 3:** The predicted plasmids of *P. mirabilis*

ID	Annotation	Genome length	GC content
JAIFRA010000038.1	*Escherichia coli* strain 2016061604 plasmid p6061604-KPC	5,420	51.40%
DAIFPI010000089.1	*Escherichia coli* strain 190 plasmid unnamed1	6,195	65.00%
DAJQUG010000355.1	*Escherichia coli* strain 190 plasmid unnamed1	6,163	65.10%
DACXYO020000033.1	*Pseudomonas aeruginosa* strain 2047 plasmid pPA2047	10,001	61.70%
JAODOZ010000037.1	*P. mirabilis* strain HFK418 plasmid pHFK418-NDM	6,343	54.20%

## DISCUSSION

*Proteus*, a typical member of the Enterobacteriaceae family, is widely distributed in nature and can be found in a wide variety of environments. They live through putrefaction and colonize the body under favorable conditions as facultative pathogens causing a variety of human infections, including wounds, eye, gastrointestinal, and urinary tract infections. As the most common pathogen in the genus, *P. mirabilis* is widely distributed in the natural environment and mammalian intestinal tract and is, therefore, worthy of in-depth study at the genomic level (43).

In this study, a pan-genomic analysis was performed based on the genome of 1,267 strains of *P. mirabilis* publicly available online, which showed a total of 2,093 core gene families, accounting for 4.76% of the total number of genes. The pan-genomic map showed an obvious open conformation, which indicates that *P. mirabilis* has a high degree of genomic plasticity and adaptability, which means that the species can adapt and respond rapidly to different environments through horizontal gene transfer, recombination, and mutation. In addition, since there is no ST typing for *P. mirabilis*, we also tried to construct cgMLST markers for this species in this study. The traditional MLST markers can be used to determine the genetic relationship between strains and compare the genetic diversity of bacteria. However, due to the limitation of the sequence length of MLST markers, they often do not have sufficient resolution to distinguish the genetic diversity of bacterial populations. CgMLST, as a novel molecular marker, can be used to identify and compare the genetic diversity of bacteria using genome-wide data rather than just the sequences of specific loci. CgMLST provides a more comprehensive and detailed analysis of the genetic diversity of bacteria and can be used to provide a higher resolution, which is more accurate than MLST (32). This new marker allows for a more complete characterization of the genetic diversity and evolutionary relationships of bacterial strains.

In recent years, with the misuse of antibiotics, there have been increasing reports of drug resistance in *P. mirabilis*, and its resistance is also on the rise (44). Based on previous studies, most of the drug resistance mechanisms of *P. mirabilis* are mainly focused on some individual cases, and multiple drug resistance mechanisms have not been evaluated at the population level. In this paper, we focus on the detailed analysis of carbapenem and quinolone resistance in *P. mirabilis*. Beta-lactam antibiotics are the most commonly used class of antibiotics in clinical anti-infective therapy, and with the widespread use of these drugs and the evolution of pathogenic bacteria, the problem of drug resistance of pathogenic bacteria has arisen. In our study, the incidence of beta-lactam resistance in *P. mirabilis* was 57.4%, and carbapenems in particular showed similarly high resistance (29.50%). In addition, the number of quinolone antimicrobial drugs in recent years has also increased year by year. In the present study, a higher frequency of quinolone resistance (*qnr* gene detection rate: 13.34% and drug-resistant loci rate: 25%) was also found in *P. mirabilis*. The prediction of plasmid sequences of *P. mirabilis* was carried out using the pre-developed tool Plasmer. In total, we found more than 8,000 plasmid fragments associated with drug resistance, which indicates that the drug resistance situation in this species has been very serious, and most of the resistance is mediated by plasmids. In particular, the presence of more than 700 plasmid fragments for quinolone resistance and carbapenem resistance suggests that *P. mirabilis* has become very severe in terms of resistance to first-line antibiotics. We filtered five complete plasmids for genomic annotation analysis and found that most of these plasmids were from common pathogens such as *Escherichia coli*, suggesting that there may be a large number of gene exchanges between *P. mirabilis* and these common pathogens, and a large number of resistance genes of this strain may also have been transferred from these common pathogens. In clinical microbiology, *Klebsiella pneumoniae*, *Staphylococcus aureus*, *Enterococcus faecalis*, and *Pseudomonas aeruginosa* have attracted widespread attention due to the presence of ultra-high levels of multi-drug resistance and are also known as “superbugs.” Our results show that the same trend of multidrug resistance exists in *P. mirabilis*, and we suggest that this species may be a new superbug that poses a serious threat to the treatment of infections.

Previous studies have shown that *P. mirabilis* can produce urease with very high activity, thus indirectly causing kidney stone formation. The structure of urease gene clusters from different bacterial sources varies, and they differ in the number and order of genes. In this paper, by studying the urease gene cluster of *P. mirabilis*, we found that the *P. mirabilis* gene cluster belongs to class I that has been reported. The structural genes for urease of *P. mirabilis* are trimeric protein structures formed by γ, β, and α subunits encoded by *ureA*, *ureB*, and *ureC* but do not possess urease activity. The auxiliary proteins encoded by the genes *ureD*, *ureE*, *ureF*, and *ureG* assist in the activation of most bacterial ureases and are encoded by the auxiliary genes *ureD*, *ureE*, *ureF*, *ureG*, respectively. The regulatory gene *ureR*, a member of the AraC/XylS family of transcriptional regulators, activates the expression of the urease gene cluster in the presence of urea (45). Urease activation is essentially a process of metal assembly of nickel ions with urease proteins. Although the lack of functional validation may limit our further accurate understanding of the function of the gene clusters in *P. mirabilis*, a screen of the urease gene clusters in the species revealed that most of the strains carry such gene cluster, suggesting the importance of this function.

### Conclusion

This study employed a comprehensive genomic approach to elucidate the notable genetic diversity of *P. mirabilis*. A cgMLST marker and evolutionary tree were established, indicating the bacteria’s adaptability and capacity to flourish in diverse environments as a consequence of its evolutionary history of mutations and adaptations.

Furthermore, the study focused on the drug resistance of *P. mirabilis*, with a specific emphasis on carbapenems and quinolones. The plasmid analysis identified resistance genes, indicating that this bacterium may prove to be a challenging target for antibiotic treatment, which could complicate infection management in clinical settings.

Finally, the urease gene cluster of *P. mirabilis* was investigated, and it was found to have a high frequency at the level of urease genes. The pervasive presence of the urease gene cluster in *P. mirabilis* underscores its pivotal role in bacterial physiology and elucidates the direct correlation between these gene clusters and urease activity, as well as their potential involvement in the formation of kidney stones.

In conclusion, the comparative genomic analysis of *P. mirabilis* in this study demonstrated the genetic diversity, drug resistance, and urease gene characteristics of this bacterium. It is therefore imperative to reinforce the prevention and control of this bacterium and the rational use of antibiotics in order to mitigate its impact on human health. Further studies on the resistance and pathogenic mechanisms of *P. mirabilis* may facilitate the development of new treatment strategies and control measures.

## Data Availability

The data set analyses during the current study are available in the NCBI database. For details, please refer to Table S2 (A).

## SUPPLEMENTAL MATERIAL

Figure S1 to S5 spectrum.00992-24-s0001.pdf Fig. S1: Detailed study design flowchart for Genomic insights on cgMLST markers, drug resistance, and urease cluster of *P. mirabilis* strains. Fig. S2: Pan-genome and core genome mapping of *P. mirabilis*. Fig. S3: The phylogenetic tree of *P. mirabilis* based on cgMLST. Fig. S4: ANI analysis map of 1,267 *P. mirabilis* genomes. Fig. S5: Single-copy consistent evolutionary tree diagram of urease from *P. mirabilis*.

Table S1 spectrum.00992-24-s0002.xlsx Public genome data analysis.

Table S2 spectrum.00992-24-s0003.xlsx (A) Metadata of *P. mirabilis* genomes. (B) 2092 gene IDs of *P. mirabilis* strain GCA_000069965.1_ASM6996v1 as reference.

Table S3 spectrum.00992-24-s0004.xlsx Beta-lactamase resistance statistics in 1267 strains of *P. mirabilis*.

Table S4 spectrum.00992-24-s0005.xlsx Analysis of quinolone resistant drugs.

Table S5 spectrum.00992-24-s0006.xlsx Drug-resistance of plasmids in *P. mirabilis*.

ASM does not own the copyrights to Supplemental Material that may be linked to, or accessed through, an article. The authors have granted ASM a non-exclusive, world-wide license to publish the Supplemental Material files. Please contact the corresponding author directly for reuse.
