# Supplementary material for: Genomic insights on cgMLST markers, drug resistance, and urease cluster of Proteus mirabilis strains
Source: Microbiol Spectr. 2024 Dec 6;13(1):e00992-24. doi: 10.1128/spectrum.00992-24 (PMC11705806; doi:10.1128/spectrum.00992-24)
Supplement: Figure S1 to S5 — Fig. S1: Detailed study design flowchart for Genomic insights on cgMLST markers, drug resistance, and urease cluster of P. mirabilis strains. Fig. S2: Pan-genome and core genome mapping of P. mirabilis. Fig. S3: The phylogenetic tree of P. mirabilis based on cgMLST. Fig. S4: ANI analysis map of 1,267 P. mirabilis genomes. Fig. S5: Single-copy consistent evolutionary tree diagram of urease from P. mirabilis. [file spectrum.00992-24-s0001.pdf]

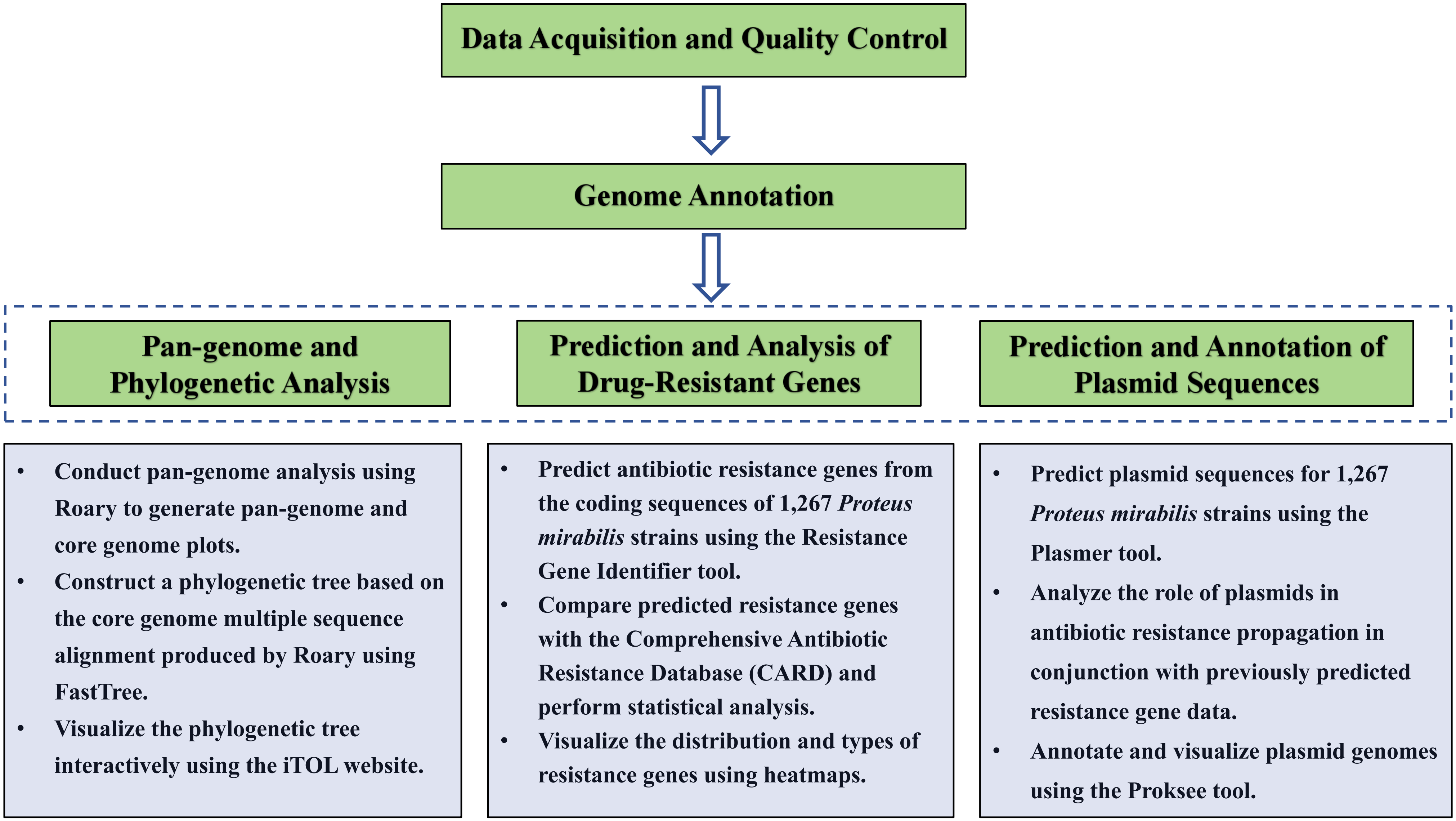

Figure S1 Detailed study design flowchart for Genomic insights on cgMLST markers, drug resistance, and urease cluster of *P. mirabilis* strains.

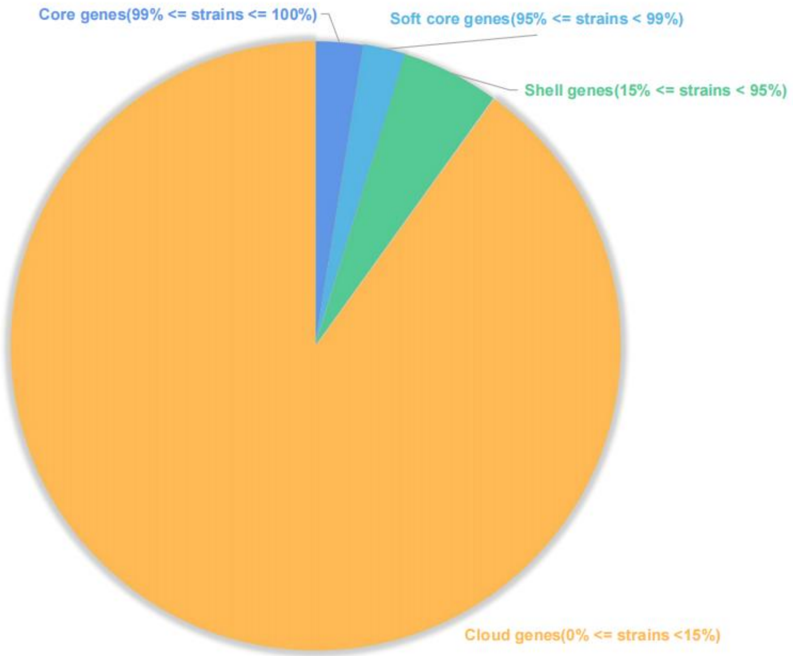

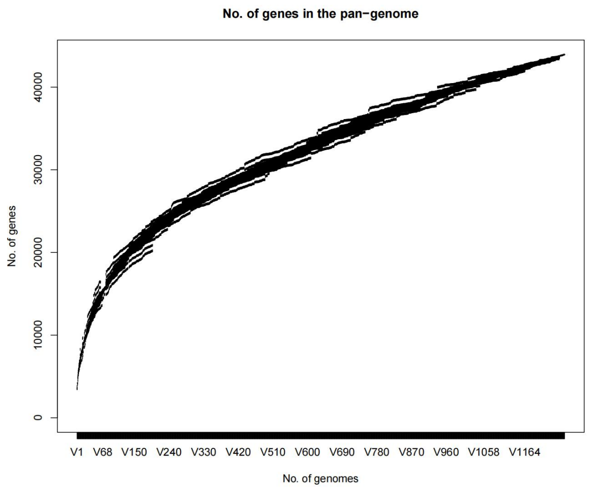

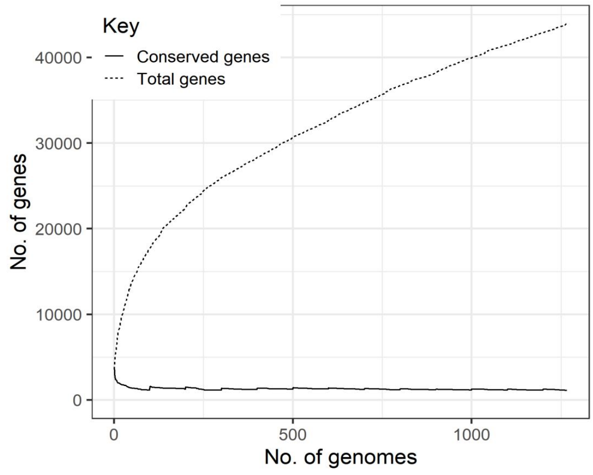

Figure S2 Pan-genome and core genome mapping of *P. mirabilis.*

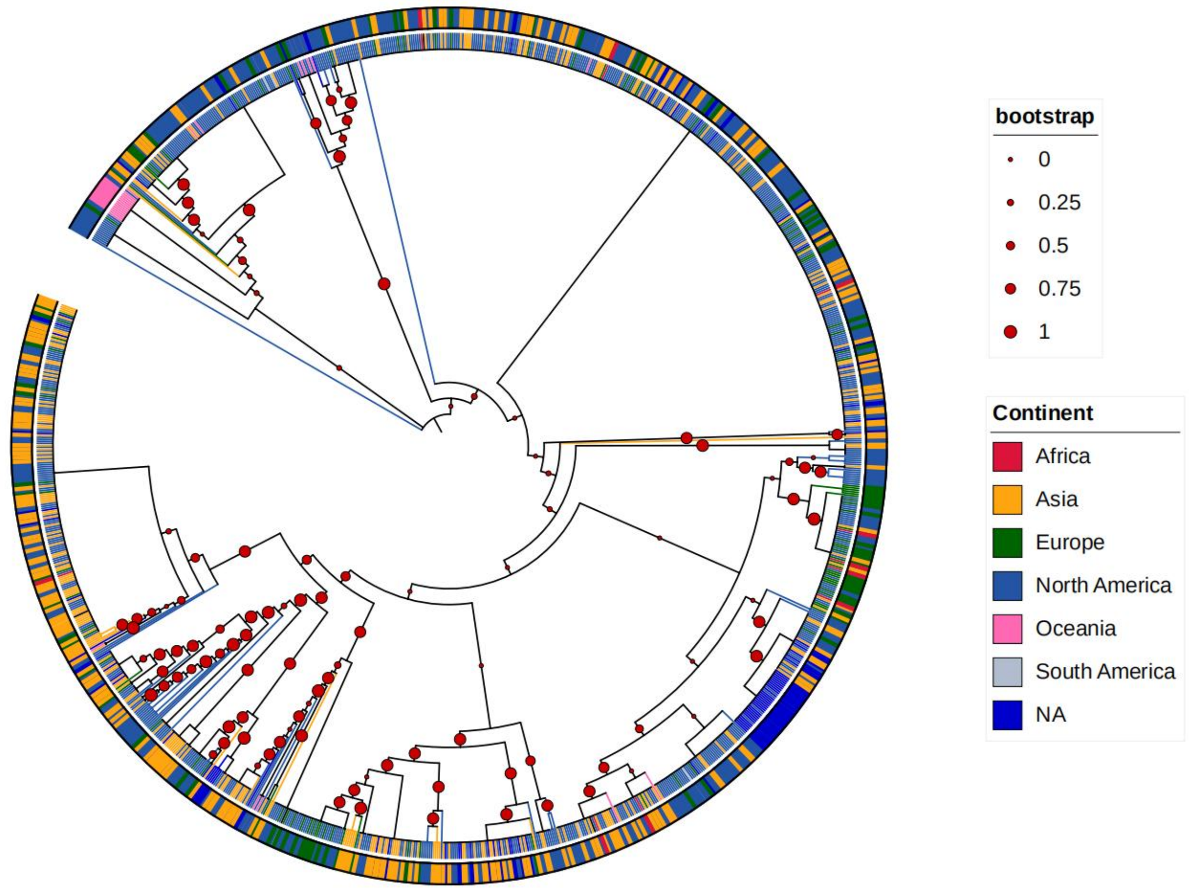

Figure S3 The phylogenetic tree of *P. mirabilis* based on cgMLST.

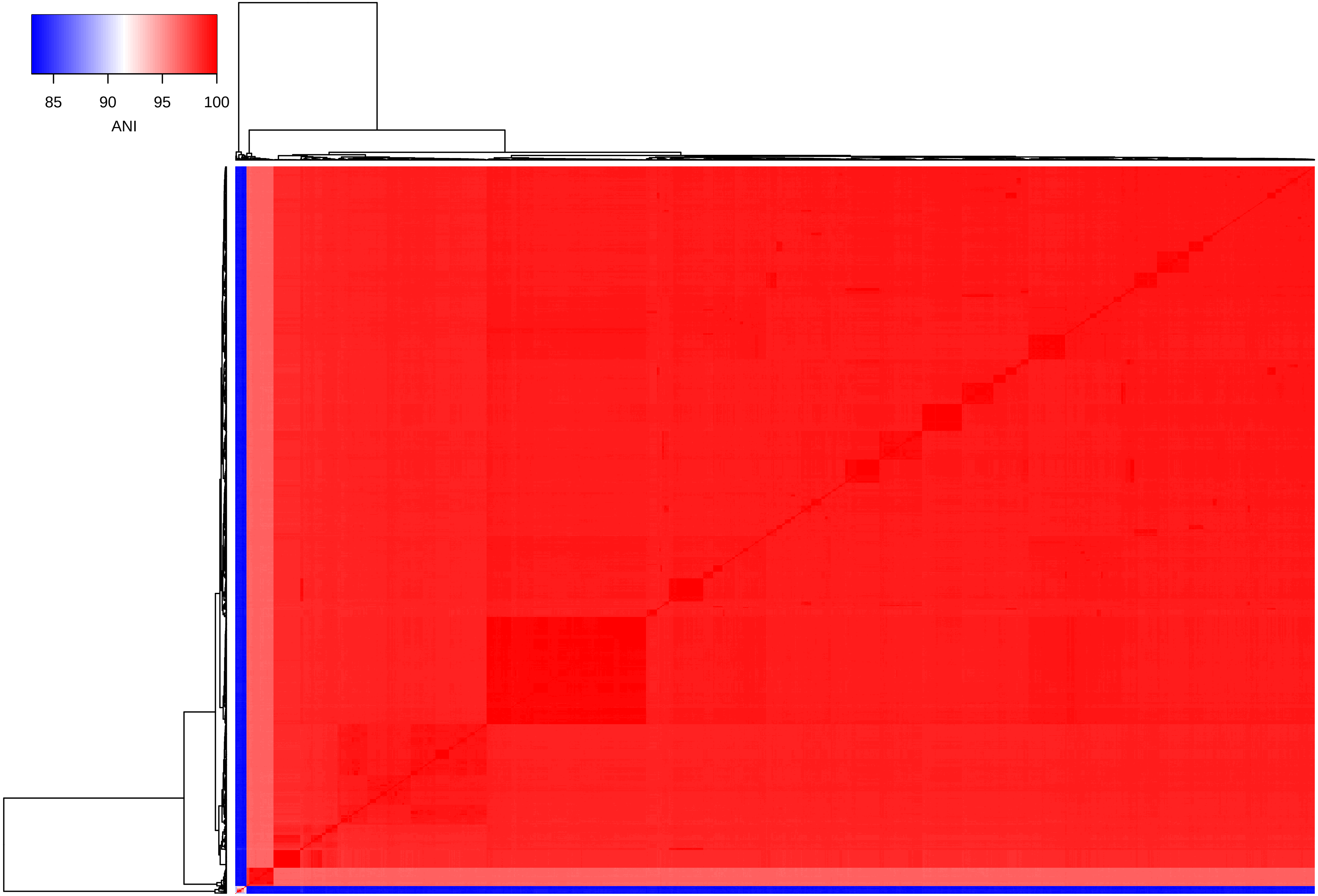

Figure S4 ANI analysis map of 1,267 *P. mirabilis* genomes.

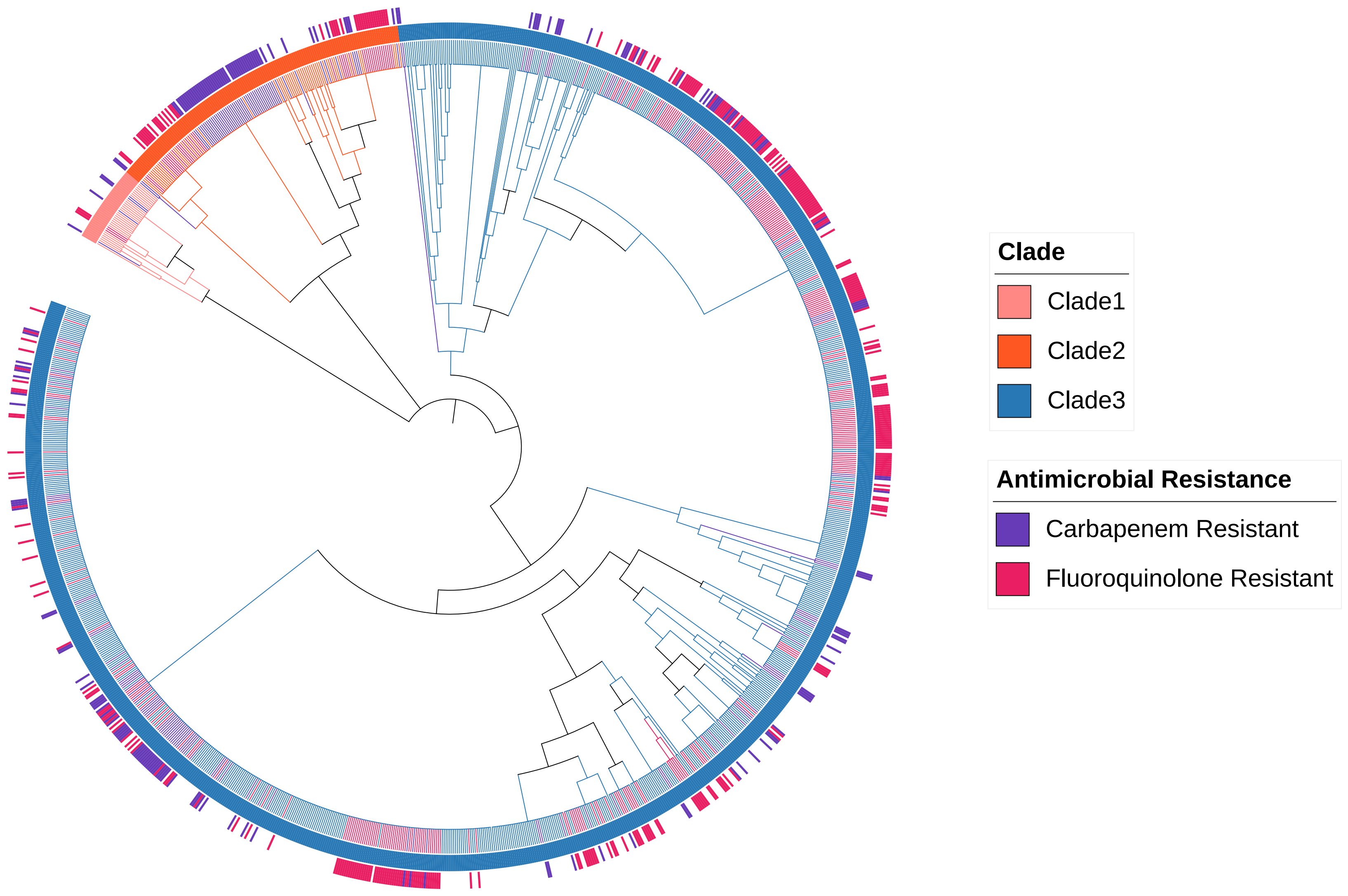

Figure S5 Single-copy consistent evolutionary tree diagram of urease from *P. mirabilis.*
